# Involvement of Synaptonemal Complex Proteins in Sex Chromosome Segregation during Marsupial Male Meiosis

**DOI:** 10.1371/journal.pgen.0020136

**Published:** 2006-08-25

**Authors:** Jesús Page, Alberto Viera, María Teresa Parra, Roberto de la Fuente, José Ángel Suja, Ignacio Prieto, José Luis Barbero, Julio S Rufas, Soledad Berríos, Raúl Fernández-Donoso

**Affiliations:** 1 Unidad de Biología Celular, Departamento de Biología, Facultad de Ciencias, Universidad Autónoma de Madrid, Madrid, Spain; 2 Programa de Genética Humana, Instituto de Ciencias Biomédicas, Facultad de Medicina, Universidad de Chile, Santiago, Chile; 3 Departamento de Inmunología y Oncología, Centro Nacional de Biotecnología, CSIC, Madrid, Spain; Stowers Institute for Medical Research, United States of America

## Abstract

Marsupial sex chromosomes break the rule that recombination during first meiotic prophase is necessary to ensure reductional segregation during first meiotic division. It is widely accepted that in marsupials X and Y chromosomes do not share homologous regions, and during male first meiotic prophase the synaptonemal complex is absent between them. Although these sex chromosomes do not recombine, they segregate reductionally in anaphase I. We have investigated the nature of sex chromosome association in spermatocytes of the marsupial *Thylamys elegans,* in order to discern the mechanisms involved in ensuring their proper segregation. We focused on the localization of the axial/lateral element protein SCP3 and the cohesin subunit STAG3. Our results show that X and Y chromosomes never appear as univalents in metaphase I, but they remain associated until they orientate and segregate to opposite poles. However, they must not be tied by a chiasma since their separation precedes the release of the sister chromatid cohesion. Instead, we show they are associated by the dense plate, a SCP3-rich structure that is organized during the first meiotic prophase and that is still present at metaphase I. Surprisingly, the dense plate incorporates SCP1, the main protein of the central element of the synaptonemal complex, from diplotene until telophase I. Once sex chromosomes are under spindle tension, they move to opposite poles losing contact with the dense plate and undergoing early segregation. Thus, the segregation of the achiasmatic T. elegans sex chromosomes seems to be ensured by the presence in metaphase I of a synaptonemal complex-derived structure. This feature, unique among vertebrates, indicates that synaptonemal complex elements may play a role in chromosome segregation.

## Synopsis

Meiosis is the fascinating kind of cell division that leads to the formation of gametes, and thus is essential for sexual reproduction. During meiosis, the part of our genome coming from our father and that coming from our mother play the last act of a love story initiated at the very moment of fertilization. This performance includes the search and close pairing of each chromosome with its homolog, the interchange of their genetic information, and their ultimate segregation into different cells. Everything occurs in an ordered and proper fashion because each chromosome is able to recognize its homolog and recombine with it. This ensures they are correctly transmitted to the next generation. But exceptions exist to this general rule. The authors show in this report that in the meiosis of the South American marsupial, *Thylamys elegans,* the sex chromosomes have the ability to segregate without undergoing meiotic recombination. The sex chromosomes take profit of the organization of a structure only present in marsupials, the dense plate, which serves the purpose of maintaining X and Y chromosomes associated until they segregate to different daughter cells. Surprisingly, this dense plate is composed of two proteins of the synaptonemal complex not meant to be at that place at that time. This finding teaches us that wildlife has plenty of way-out species, most of them unexplored, which challenge our accepted paradigms.

## Introduction

Segregation of homologous chromosomes is one of the key events of the meiotic process and is perhaps the only universally conserved feature throughout the evolution of meiosis in all eukaryotes. During the first meiotic division, homologous chromosomes, as a rule, migrate to opposite poles of the dividing cell, leading to the reduction of ploidy. For this segregation to occur properly, at least two conditions are necessary: (i) homologous chromosomes must orientate syntelically, i.e., both sister kinetochores must be closely associated and facing the same pole and (ii) homologs must be tightened until the onset of anaphase.

The way in which the first condition is accomplished has been a matter of classical study. It has been demonstrated that although sister kinetochores are already duplicated when chromosomes enter first meiotic division, they behave as a single functional unit [1–5]. This special behavior is a specific feature of the kinetochores during the first meiotic division [6], which could rely on the existence of meiosis-specific components that prevent the separation of sister kinetochores by establishing a sort of sister kinetochore cohesion during meiosis-I [7]. One of these components, monopolin, has been described in budding yeast [8]; and other mechanisms, mainly involving cohesin subunits, have been found in fission yeast, insects, and mammals [7,9,10].

The second requisite, the maintenance of homologous chromosome association, depends almost universally on the occurrence of homologous recombination. Reciprocal recombination leads to the formation of crossovers between homologs, which after the disorganization of the synaptonemal complex (SC) are visualized as chiasmata. The occurrence of at least one reciprocal recombination event is required in order to hold homologous chromosomes associated from the disorganization of the SC until the onset of anaphase I, when homologs segregate. In recent years, it has been clarified that chiasmata per se do not maintain homolog association, but this is due to the presence of mechanisms that maintain the cohesion between sister chromatids [11,12]. Many of the components involved in sister chromatid cohesion in mammalian meiosis have been identified in recent years. These include the SCP3 protein, a component of the lateral element (LE) of the SC [13–15] and a series of proteins that participate in the formation of one or several cohesin complexes: SMC1α, SMC1β, SMC3, STAG3, REC8, and RAD21 [7,16–19].

According to this model, recombination ensures accurate chromosome segregation during first meiotic division. However, in a wide variety of organisms, flies and butterflies for instance, recombination between homologous chromosomes is absent in one of the sexes, and alternative mechanisms to ensure proper meiotic segregation have been developed [20–23]. Among mammals, the most widespread case of achiasmatic meiosis is found in the sex chromosomes of marsupial males, in which the X and Y chromosomes do not share a homologous region [24,25]. Accordingly, sex chromosome association during the first meiotic prophase is asynaptic, i.e., there is no formation of a SC central element (CE) between their axial elements (AEs). Instead, their association is maintained by a marsupial specific structure called the dense plate (DP) [26–29] developed from sex chromosomal AEs [30,31].

It is well established that the DP maintains sex chromosome association up to the end of the pachytene stage of the first meiotic prophase [26–30]. However, it is not clear what mechanism is responsible for maintaining this association in later stages of prophase I and metaphase I. In a seminal work on marsupial meiosis, Koller [32] indicated that sex chromosomes appear regularly associated in metaphase I. Koller and other authors thereafter, postulated that one chiasma must exist between sex chromosomes in order to ensure their segregation [26,32,33]. However, according to the currently reported absence of homology and synapsis between sex chromosomes, recombination and thus chiasma formation should not occur. In this sense, some authors have reported a high frequency, up to 50%, of sex chromosome univalents in metaphase I in some species [34]. Noticeably, these data are in striking discrepancy with the results previously reported in the same species [32].

On these grounds, the nature of the mechanism that ensures the proper segregation of sex chromosomes in marsupial male meiosis remains controversial. In this report we present a detailed analysis of the meiotic behavior of sex chromosomes during the first meiotic division in the South American marsupial *Thylamys elegans.* In order to search for plausible mechanisms of chromosome association we have studied the location of SCP3 and SCP1 proteins of the SC and the meiotic cohesin subunit STAG3, paying special attention to their cycle of appearance and release from sex chromosomes. We also contrasted the hypothesis of a telomeric association of sex chromosomes through the location of telomeric sequences. Our results indicate that the persistence of sex chromosome association by the DP developed in pachytene is the most plausible mechanism to ensure sex chromosome segregation during first meiotic division, and there is no evidence for a chiasmatic or telomeric association.

## Results

The karyotype of T. elegans is composed of six pairs of autosomes (three submetacentric, one metacentric, and two acrocentric) and a pair of sex chromosomes (XX females, XY males), both X and Y being submetacentric [30,35,36]. In order to accurately analyze the orientation and segregation of chromosomes during both meiotic divisions, we have used a squash technique that preserves the native organization of chromosomes within the cell.

### Segregation of Sex Chromosomes

**Figure 1:**
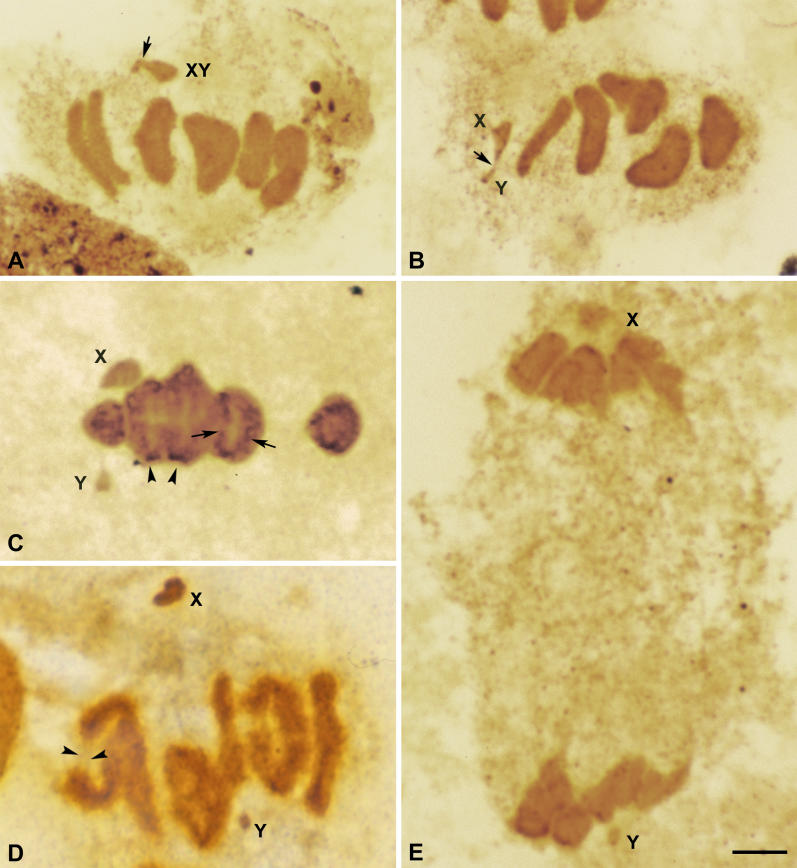
Silver Staining of T. elegans Spermatocytes (A–C) Metaphase I. The autosomal bivalents appear aligned at the cell equator. (A) In an early stage, sex chromosomes (X, Y) appear associated out of the metaphase I plate, near one of the cell poles. There is a wide region of contact between both sex chromosomes (arrow). (B) In a middle stage, sex chromosomes (X, Y) appear situated at the cell equator, each facing a cell pole. The chromatin that connects both sex chromosomes appears stretched (arrow). (C) In a late stage, sex chromosomes (X, Y) have initiated their segregation, while the autosomes are still stabilized at the metaphase plate. Note that in this favorable case, silver staining has revealed the kinetochores of chromosomes (arrowheads) and linear elements inside the chromatin (arrows). (D) Early anaphase I. Autosomes initiate their segregation. Chromatin bridges are seen between the telomeres of some autosomes (arrowheads). Sex chromosomes (X, Y) appear at opposite poles. (E) Telophase I. Each sex chromosome (X, Y) is incorporated to one pole. Bar: 5 μm.

The process of orientation and segregation of chromosomes was first followed using silver staining on squashed spermatocytes (Figure 1). The first remarkable feature in relation to sex chromosomes is that they appear associated to each other at metaphase I (Figure 1A–1C). At this stage all autosomal bivalents are aligned at the cell equator. However, sex chromosomes undergo a sequence of changes in their morphology and orientation. Since these changes occur while the autosomes remain clearly orientated and stabilized at the metaphase plate, we have subdivided metaphase I stage into early, mid, and late as regards sex chromosome behavior. In early metaphase I, sex chromosomes are not bioriented, but instead they are located outside the cell equator and close to one of the cell poles (Figure 1A). In a subsequent stage (mid metaphase I), the sex pair seems to achieve a bipolar orientation and move to the equator of the cell, each chromosome facing to a different cell pole (Figure 1B). During this phase the chromosomes are still connected, although the chromatin is stretched at the contact region. We have observed that during this process of biorientation the sex pair often remains closer to the pole to which X is facing. The biorientation of sex chromosomes is followed by a stage (late metaphase I) in which they lose their contact, and each one moves to an opposite pole (Figure 1C). Since autosomal bivalents still remain stabilized at the metaphase I plate at this stage, it is clear that the segregation of sex chromosomes precedes the segregation of autosomes. At the onset of anaphase I (Figure 1D), while autosomes initiate their segregation, sex chromosomes are usually near the poles. The first meiotic division is always reductional, both for autosomes and sex chromosomes (Figure 1E). We have never observed lagging sex chromosomes.

### Localization of STAG3 Cohesin Subunit

**Figure 2:**
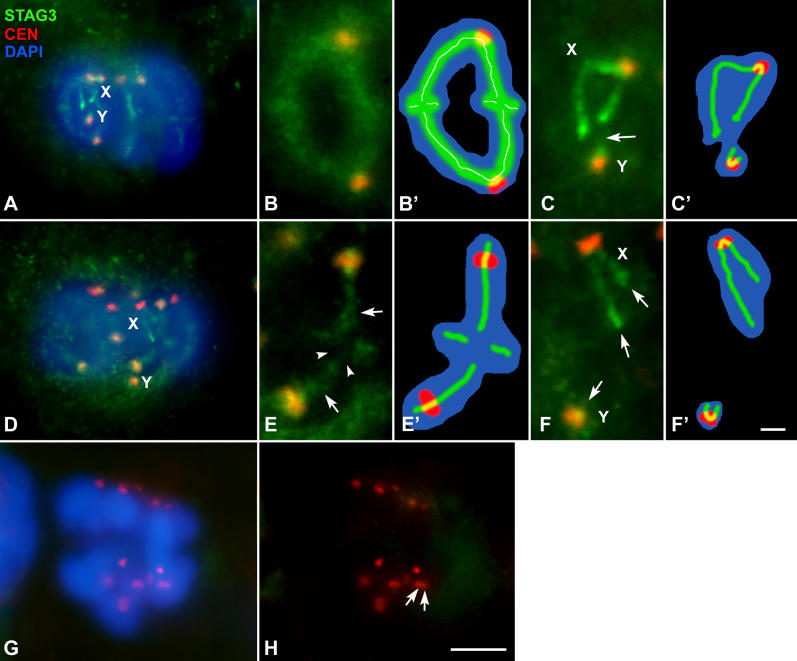
Immunolabeling of Spermatocytes with Anti-STAG3 (Green) and Anti-Centromere (Red) Antibodies and Staining of the Chromatin with DAPI (Blue) Several focal planes have been superimposed and projected in a single plane in each image. (A–C) Early–middle metaphase I. All bivalents are aligned at the metaphase I plate. Sex chromosomes (X, Y) also appear at the cell equator and their centromeres oriented to opposite poles. Note that the position of the sex pair is not equidistant from the poles. Instead, the X chromosome seems to be closer to the pole than the Y chromosome. (B) An enlarged autosomal bivalent of the same cell in which STAG3 appears as diffuse lines at the interchromatid domain. (B') Representation of the same bivalent shown in (B). Sex chromosomes are in close proximity, but there is no contact between the STAG-labeled structures (arrow). (C') Schematic representation of the sex chromosomes in (C). (D–F) Late metaphase I. Autosomal bivalents are still stabilized at the cell equator. STAG3 labeling is detectable in both autosomes and sex chromosomes (X, Y). (E) Enlargement of an autosomal bivalent with a subdistal chiasma in the long arm. The signal of STAG3 runs along the interchromatid domain (arrows) but interrupts at the chiasma site (arrowheads). (E') Schematic representation of the same bivalent shown in (E). Sex chromosomes (detailed in F) have initiated their segregation, even though the STAG3 labeling is still present along their interchromatid domains; arrows in (F). (F') Representation of the sex chromosomes in (F). (G and H) Anaphase I. Homologous chromosomes start to segregate to opposite poles. The STAG3 labeling is completely absent from both autosomes and sex chromosomes. The centromere signals in most chromosomes are seen as double dots (arrows), corresponding to sister kinetochores. Bars: 5 μm in (A), (D), (G), and (H); 1 μm in (B), (C), (E), and (F).

The fact that sex chromosomes do not appear as univalents in metaphase I suggests that there must be a mechanism ensuring their association. We first tested the hypothesis of a chiasmatic association. For this purpose we followed the localization of STAG3, a subunit of the meiotic cohesin complex (Figure 2). The location of this protein during prophase I is similar to that described in mouse [17,31] and has been previously described in detail [31]. In metaphase I this protein appears associated to the bivalents in the region of contact between the sister chromatids of each chromosome, in the so-called interchromatid domain [9] (Figure 2A–2E). The signal of STAG3 is detected as a diffuse line that runs all along the internal region of autosomes but interrupts at the points of chiasma (Figure 2E). In the sex chromosomes STAG3 also appears at the interchromatid domain, running regularly from one chromosome end to the other (Figure 2C and 2F). There is no connection between the signals of both chromosomes. The axial structure labeled by STAG3 on the sex chromosomes is present in early metaphase I, when they are still associated (Figure 2C–2C'), and also in mid and late metaphase I when sex chromosomes initiate their segregation and separate from each other (Figure 2F–2F'). During this later stage, autosomal bivalents remain stabilized in the metaphase I plate with the STAG3 signal present at their interchromatid domains. The onset of anaphase I, in which homologous chromosomes initiate their segregation, is concomitant with the complete disappearance of the STAG3 signal, both in the autosomes and sex chromosomes (Figure 2G–2H), as expected for a protein involved in the maintenance of sister chromatid arm cohesion, and following the same pattern described for mouse [17]. STAG3 is not present during second meiotic division. The separation of sex chromosomes preceding the release of sister chromatid cohesion strongly supports the idea that the association of these chromosomes is not mediated by a chiasma.

### Localization of Telomeric Sequences

**Figure 3:**
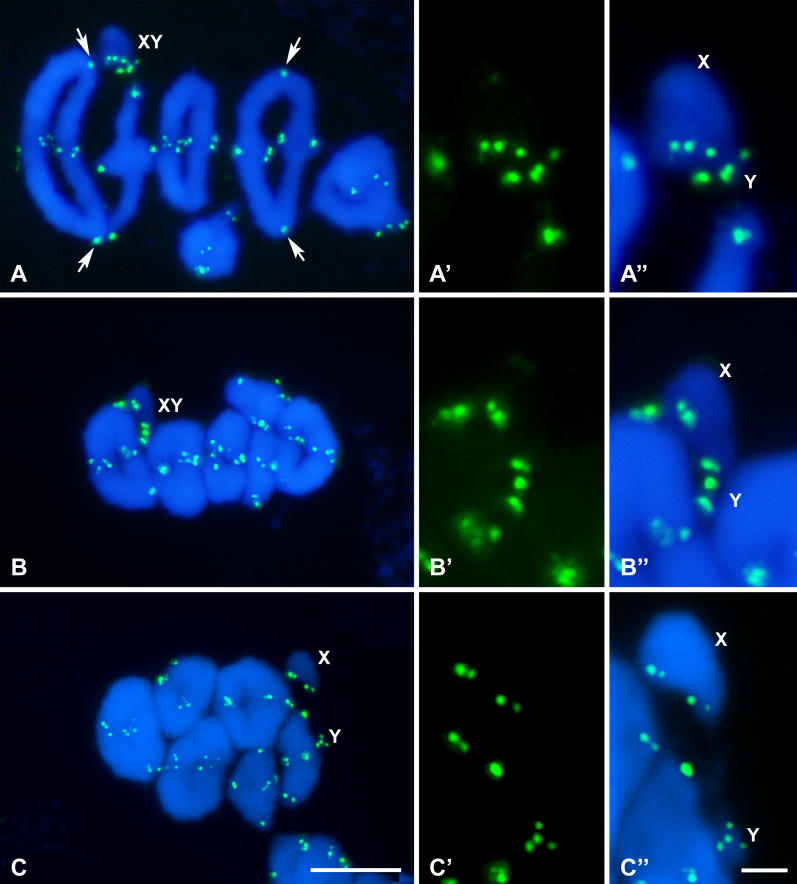
FISH of Telomeric Repeats (Green) and Chromatin Staining with DAPI (Blue) (A–A'') Early metaphase I. All chromosomes show distal telomeric signals, and two large bivalents also show a hybridization signal near the centromeric region (arrows). (A') and (A'') show an enlargement of sex chromosomes in the cell shown in (A). Sex chromosomes (X, Y) are associated, each one facing a cell pole, but displaced from the cell equator. The telomeric repeats of the X and Y appear in close apposition, but without contact. (B–B'') Mid metaphase I. The sex pair is still located out of the cell equator. At this stage, the sex chromosomes show clear signs of being under spindle tension, but their association is not mediated by telomeric repeats, since the distal telomeric signals of both sex chromosomes, although being in close proximity, are not in contact. (C–C'') Late metaphase I. Sex chromosomes have already initiated their segregation, while the autosomes are still stabilized on the metaphase I plate. No contact between telomeres of the sex chromosomes is detected. Bars: 5 μm in (A–C); 1 μm in (A'–C'').

In order to analyze whether telomeric sequences could be involved in sex chromosome association, we performed the localization of the (TTAGGG)n telomeric repeats by means of fluorescent in situ hybridization (FISH) (Figure 3). Telomeric DNA signals appeared, as expected, at the ends of all chromosomes. In most cases eight terminal signals were detected in each bivalent, although in some cases the signals of the sister chromatids appeared so close to each other that they were seen as a single signal. There are two autosomal bivalents that bear telomeric repeats at the centromeric regions (Figure 3A). These interstitial repeats have been described in a variety of species, including marsupials, and are thought to represent remnants of Robertsonian fusions [37–40].

Telomeric repeats of each sex chromosome are clearly distinguished and individualized. It is possible to observe four signals on the X chromosome and four on the Y chromosome (Figure 3A–3A''). As it occurs in the autosomes, the signals on the sister chromatids can be sometimes overlapped (Figure 3B–3B''), but we never found association between the telomeric repeats of both sex chromosomes.

As described above, there is a sequence of orientation of the sex pair during metaphase I. In a first stage, corresponding to the positioning of the sex chromosomes out of the metaphase I plate, the eight telomere signals seem to be clustered, although individualized (Figure 3A'–3A''). When the sex pair reaches a bipolar orientation, the proximal telomeres, i.e., those on the short arms of both chromosomes, separate from this cluster, but distal telomeres of the X and Y chromosomes remain close to each other without any contact (Figure 3B'–3B''). Finally, sex chromosomes separate completely, initiating their migration to opposite poles, while autosomes remain at the metaphase I plate. There is no connection between the telomeric repeats of the sex chromosomes at this stage (Figure 3C–3C''). This behavior seems to indicate that in the sequence of segregation of sex chromosomes, the ends of both chromosome arms could be involved in the association of the sex chromosomes in a first stage, but as soon as the spindle forces pull the chromosomes to the poles, the short arms are the first ones to lose contact and subsequently the long arms lose contact as well. Nevertheless, we have never observed any specific association of the telomeric DNA that could be responsible for maintaining the association of the sex chromosomes, since they were always clearly segregated.

### Localization of Synaptonemal Complex Components

**Figure 4:**
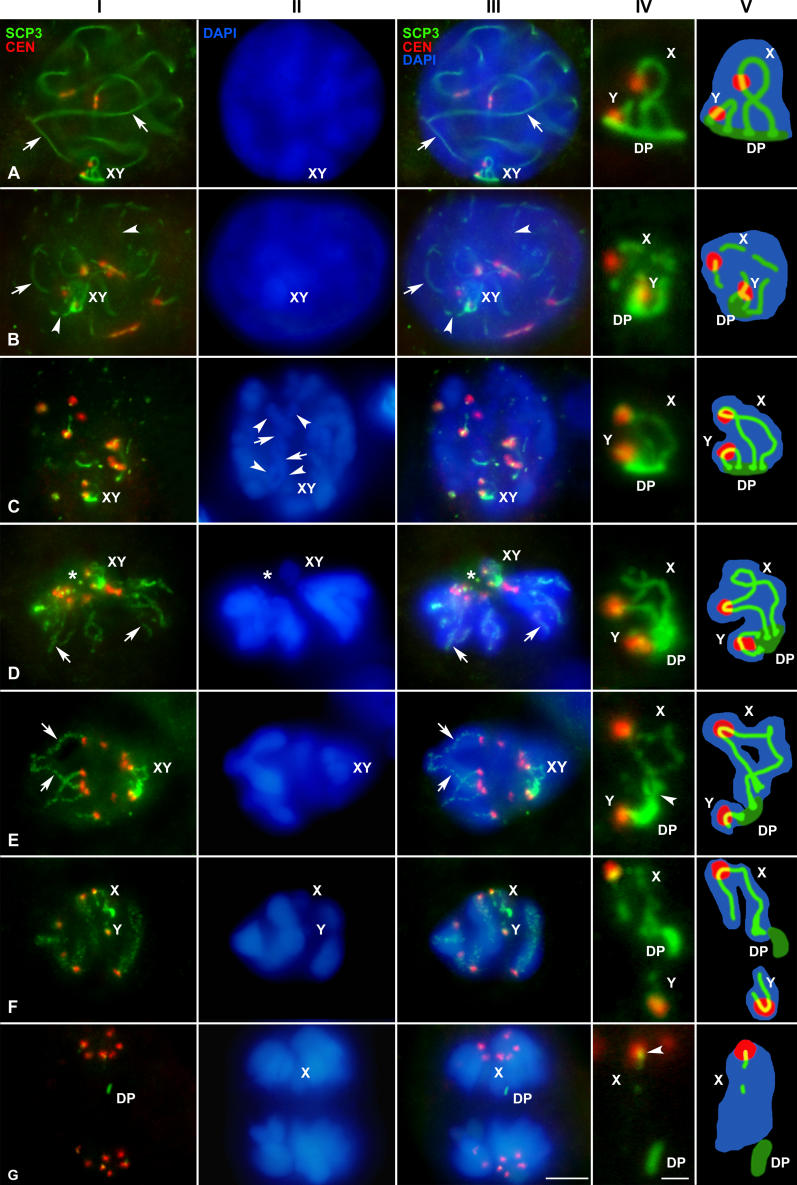
Immunolocalization of SCP3 (Green) and Centromeres (Red) and Chromatin Staining with DAPI (Blue) Several focal planes are superimposed and projected in a single plane in each picture. Column I: SCP3 and centromere signals. Column II: DAPI staining. Column III: merge of columns I and II. Column IV: detail of the sex chromosomes. Column V: schematic representations of the sex chromosomes shown in the column IV. The DP has been represented in dark green in order to differentiate it from sex chromosomal axes. (A) Pachytene. SCP3 appears as continuous lines along each autosomal bivalent (arrow) and also depicts the trajectory of sex chromosomal AEs and the DP. (B) Diffuse stage. SCP3 appears as continuous (arrow) or discontinuous lines (arrowheads) in both autosomal LEs and sex chromosome AES, and also on the DP. (C) Diplotene. The autosomal LEs appear fragmented. Homologous chromosomes appear separated (arrowheads) except at the chiasma points (arrows). However, the AEs of sex chromosomes (X, Y) appear continuous, and the labeling of the DP is also visible. (D) Prometaphase I. All centromeres appear polarized to a specific point (asterisk) that presumably corresponds to the position of the centrosomes. SCP3 remains associated with the chromosomes at the interchromatid domain (arrows). This labeling has a dotted-like appearance on the autosomes, but is continuous on the sex chromosomes (X, Y) and the DP. The tips of both sex chromosomes are in contact with the DP. (E) Early metaphase I. The SCP3 labeling is similar to previous stages, with this protein located at the interchromatid domain of all chromosomes (arrows). The bivalents have adopted a bipolar orientation with the homologous centromeres facing opposite spindle poles. The sex pair (X, Y) appears also bioriented. Both sex chromosomes remain in contact with the DP, but the short arm on the X chromosome appears detached from this structure (arrowhead). (F) Late metaphase I. Autosomal bivalents appear stabilized at the metaphase I plate. The SCP3 labeling is present at the interchromatid domain in both autosomes and sex chromosomes (X, Y). Sex chromosomes appear separated at this stage. They have lost contact, and each one has initiated its segregation to a different pole. The DP remains associated in this case to the X chromosome long arm. (G) Anaphase I. The SCP3 labeling disappears at the beginning of anaphase I from both autosomes and sex chromosomes. Only a SCP3 labeling is detected in some centromere regions (arrowhead) and on the DP. The position of the X chromosome is detectable because the DP remains associated in this case to this chromosome. Bars: 5 μm in column I–III; 1 μm in column IV.

The association of sex chromosomes during prophase I is mediated by a structure, the DP, which is formed, at least in part, by components of the LEs of the SC [30]. Thus, this structure could be a good candidate to maintain sex chromosome association in later stages. In order to test this hypothesis, we studied the localization of SCP3, a component of the SC LEs (Figure 4). During pachytene, the DP develops as a flat structure intensely stained by SCP3 at the region of association of sex chromosomal AEs to the nuclear periphery (Figure 4A). Pachytene is followed by a diffuse stage characterized by a partial decondensation of the chromatin and the fragmentation of the autosomal SCs, as well as the sex chromosome AEs (Figure 4B). During this stage the DP appears more diffuse and intermingled with the partially loosened AEs of sex chromosomes. At the beginning of diplotene, chromatin recondenses and homologous chromosomes begin to separate, remaining associated at the chiasmata points (Figure 4C). SCP3 appears along the bivalents as short threads in those regions where desynapsis has not been completed, and is absent from those where homologous chromosomes are already separated (Figure 4C). This sequence of disassembly of SCP3 departs from the pattern described for eutherian mammals, in which the LEs of the SC are maintained along the entire length of the chromosomes in diplotene [13]. During diplotene, the sex chromosomes appear associated to each other at the periphery of the nucleus, and contrary to the autosomes, their AEs are labeled with SCP3 as well defined and continuous lines (Figure 4C). The DP is also well organized and remains associated to the ends of sex chromosomes axes.

At prometaphase I all bivalents adopt a characteristic ring configuration; with all the centromeric regions polarized to a point in the protoplasm (Figure 4D). Such a polarization has been also observed in mouse spermatocytes and has been explained as being the result of a late separation of the duplicated centrosomes. Thus, the organization of a bipolar spindle takes place only after the breakdown of the nuclear envelope and not during prophase as it occurs in mitosis [41]. After this initial step, bivalents begin to congregate at the equator of the cell forming the metaphase I plate, each homolog facing towards a pole, i.e., reaching a syntelic orientation (Figure 4E and 4F). SCP3 labeling appears on the autosomal bivalents as a dotted line located at the interchromatid domain. In some bivalents it can be clearly seen that the SCP3-labeled lines of each homolog converge at certain points, corresponding to chiasma location, and the SCP3 signal is interrupted at these points (Figure 4E).

The analysis of the behavior of the sex chromosomes during prometaphase I and metaphase I shows that these chromosomes display a series of special features. They are located near the confluence point of all centromeres during early prometaphase I. In sex chromosomes, SCP3 immunostaining yields a continuous linear labeling which, as in the autosomes, appears located internally into the chromatin, most likely on the interchromatid domain (Figure 4D). This SCP3 line runs from one telomere to the other, passing through the centromere. The axial structures of the X and Y chromosome end in a curved plate structure of about 1 μm, the DP, which in this stage is reduced in size. As metaphase I proceeds, while autosomal bivalents appear orientated in the bipolar spindle, sex chromosomes remain associated occupying a peripheral position. Later, they congress to the metaphase I plate. However, at this stage we detected some changes in the nature of the association of sex chromosomes. Although the labeling of SCP3 remains along the sex chromosomes and at the DP, the short arms of both sex chromosomes lose contact with the DP (Figure 4E). In a later stage, sex chromosomes separate definitively and start to move to opposite poles (Figure 4F). This separation is coupled to the losing of contact between the sex chromosome axial structures and the DP, which may remain associated to the axis of either of the sex chromosomes, or more frequently, may locate on the protoplasm of the cell without contact with any chromosome. In any of these cases, we found that once detached from sex chromosomes the DP does not contain DNA, as revealed by DAPI staining.

**Figure 5:**
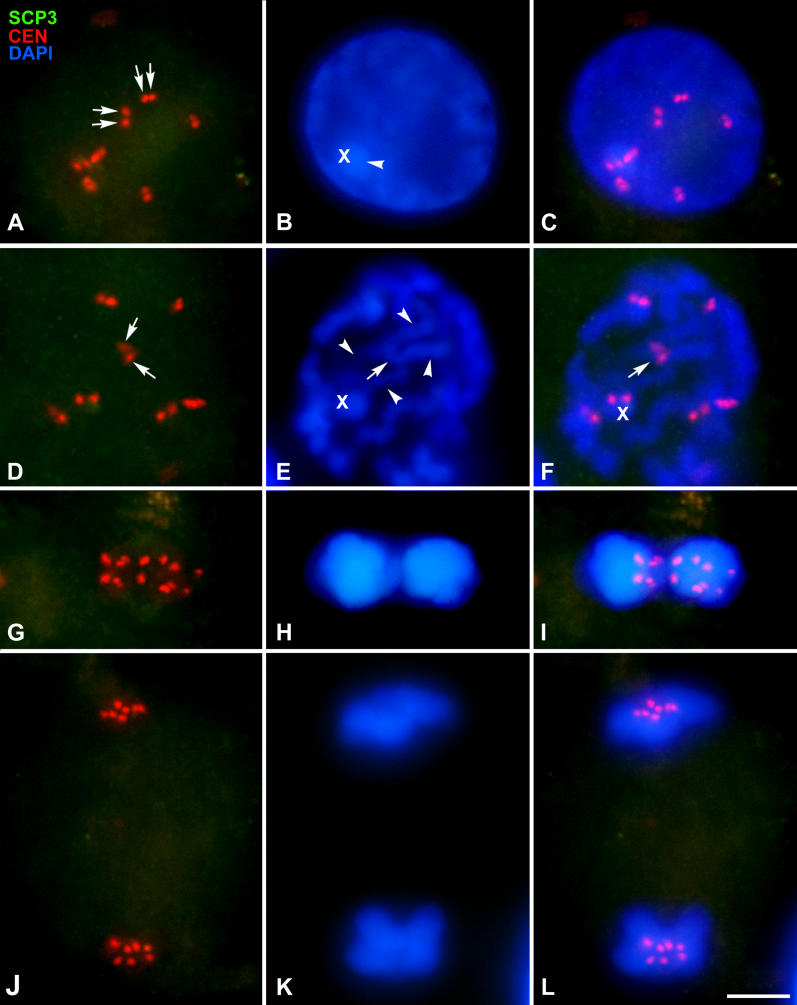
Immunolocalization of SCP3 (Green) and Centromeres (Red) and Chromatin Staining with DAPI (Blue) Several focal planes are superimposed and projected in a single plane in each picture. (A–C) Interkinesis. Seven centromeric signals are detected. Each centromere shows two signals corresponding to sister kinetochores (arrows). No SCP3 labeling is detected at all, indicating the absence of this protein during this and subsequent stages. DAPI staining (B–C) reveals a more condensed chromatin mass inside the nucleus (arrowhead) that presumably corresponds to the X chromosome. (D–F) Prophase II. The seven centromeric signals are dispersed through the nucleus and the chromatin starts to condense. Chromosomes are individualized, showing their chromatids joined only at the centromeric region (arrows) while the chromatid arms are separated (arrowheads). The putative position of the X chromosome is indicated (X). (G–I) Metaphase II. All chromosomes are aligned at the metaphase II plate. Note that the distance between sister kinetochores is greater than in prophase II. (J–L) Anaphase II. All chromosomes separate their chromatids during this stage. Seven single centromeric signals are detected at each pole. Bar: 5 μm.

During the initial steps of sex chromosome segregation, autosomal bivalents remain stabilized on the metaphase I plate and the SCP3 labeling is maintained without changes. However, at the onset of anaphase I, the SCP3 signals disappear from both the autosomes and the sex chromosomes (Figure 4G). Only a weak labeling of SCP3 persists in some centromeric regions, which are now seen as double signals with the anti-centromere serum, and also in the DP. The SCP3 labeling disappears definitively from the chromosomes during telophase I and is completely absent during interkinesis and throughout all stages of the second meiotic division (Figure 5), in agreement with the absence of SCP3 during the second meiotic division in mouse [7].

**Figure 6:**
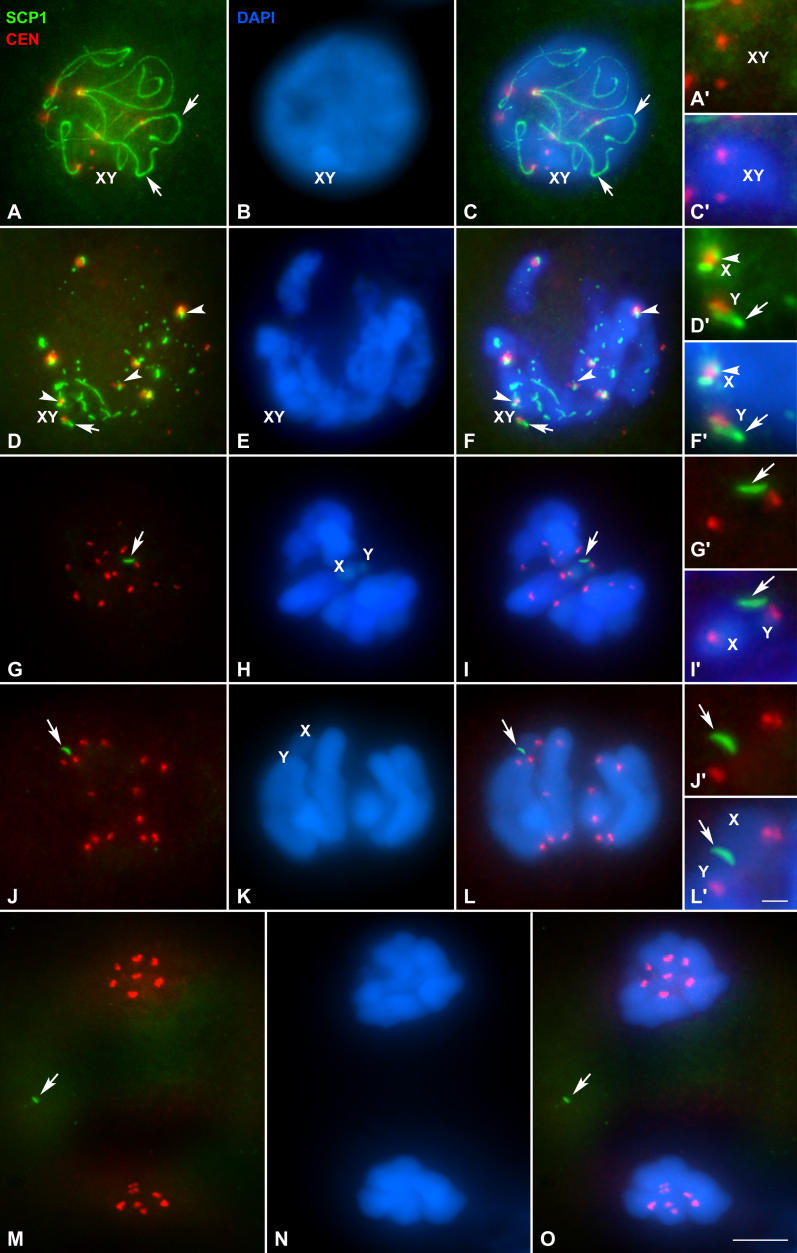
Immunolocalization of SCP1 (Green), Centromeres (Red), and Staining of the Chromatin with DAPI (Blue) Several focal planes are superimposed and projected in a single plane in each picture. (A–C) Pachytene. SCP1 appears as continuous lines along autosomes and is completely absent on the sex chromosomes. (A' and C'). Detailed view of the sex chromosomes in the same cell. (D–F) Diplotene. SCP1 mostly appears as fragmented lines that run along chromosomes. These lines are continuous in some regions. SCP1 remains associated at the centromeric regions of autosomes and also appears associated to the X chromosome centromere (arrowheads). A structure labeled with SCP1 appears at the periphery of the sex body (arrow). (D' and F') At a higher magnification this structure is clearly detected near the centromere of the Y chromosome. (G–I) Prometaphase I. SCP1 signal has completely disappeared from the chromosomes. However, a small structure is clearly labeled (arrow). Bivalents are not oriented at the metaphase plate. Sex chromosomes (X, Y) are clearly discernible, and they remain associated. (G' and I'). Enlargement of the sex chromosomes. The SCP1-labeled structure appears at the region of contact between sex chromosomes (arrow). (J–L) Metaphase I. The SCP1-labeled structure is present in the region where sex chromosomes (X, Y) are located. (J'–L') At a higher magnification it can be seen that the SCP1-labeled structure (arrow) is associated to the sex chromosomes. (M–O). Anaphase I. Homologous chromosomes have segregated and the SCP1-labeled structure is still visible (arrow). However, it does not maintain any association with the chromatin masses near the cell poles. Bar: 5 μm in (A–O); 1 μm in (A'–L').

We have also studied the localization of SCP1, a component of the CE and the transverse filaments of the SC [42] (Figure 6). The localization pattern of this protein during prophase I (zygotene to pachytene) has been described in T. elegans [30]. SCP1 appears in the regions where homologous chromosomes have synapsed, but is absent from the sex chromosomes (Figure 6A–6C). In diplotene SCP1, labeling appears as fragmented lines along the autosomal bivalents and as short lines at the centromeric regions of autosomes (Figure 6D–6F), resembling the pattern described for SCP3. Interestingly, SCP1 also appears at the centromeric region of the X chromosome and at the region of association of the sex body to the nuclear periphery. SCP1 subsequently disappears along the autosomes during diakinesis, remaining only detectable at the centromeric regions (not shown) and is completely absent from autosomes in later stages (Figure 6G–6O). Nevertheless, a small structure intensely labeled with antibodies against SCP1 is invariably found in prometaphase I (Figure 6G–6I) and metaphase I (Figure 6J–6L). This structure is associated with the sex chromosomes, and its morphology, size, and pattern of localization are identical to those of the DP labeled with SCP3. It is a small, about 1-μm long, and slightly curved structure that localizes in the region of association of sex chromosomes before their segregation, and after their separation it may either remain associated to either of the sex chromosomes, or most often randomly distributed within the protoplasm (Figure 6M–6O).

**Figure 7:**
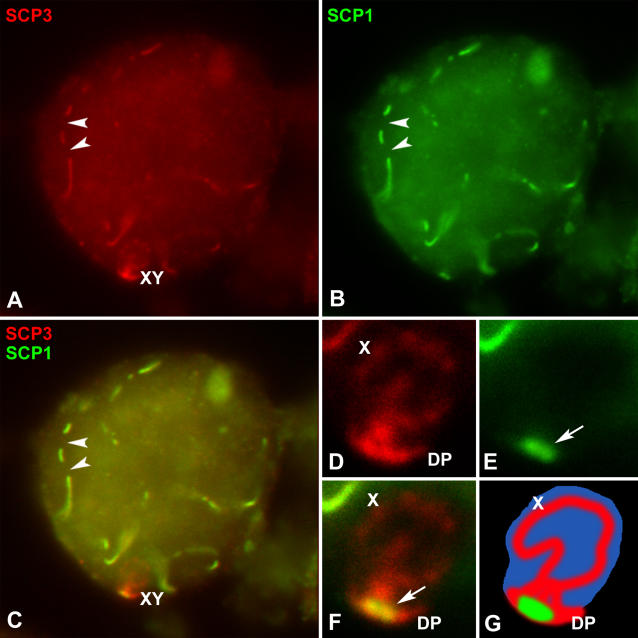
Immunolocalization of SCP3 (Red) and SCP1 (Green) and Counterstaining with DAPI (Blue) in a Spermatocyte at Diplotene Two focal planes are superimposed in each picture. (A) SCP3 lines representing the autosomal SC appear discontinuous (arrowheads). The sex chromosomes (X, Y) (enlarged in D–F) appear associated and the DP is detectable. (B) SCP1 follows a similar pattern to that of SCP3 on the autosomes, appearing as fragmented lines. (C) Merge of SCP3 and SCP1 signals, which co-localize in the autosomes but not in the sex chromosomes. (D) Enlargement of the sex chromosomes. SCP3 appears along the AEs of the sex chromosomes and also in the DP. (E) SCP1 does not appear in the AEs of sex chromosomes, but is present in the DP (arrow). (F) Merge of the signals shown in (D) and (E). Note that the distribution of SCP1 in the DP is more restricted at this stage than that of SCP3. (G) Schematic representation of the sex chromosomes.

**Figure 8:**
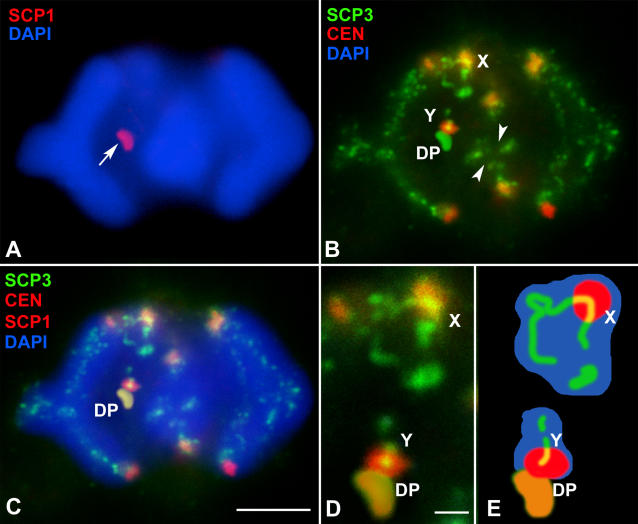
Triple Immunolocalization of SCP3 (Green), SCP1 (Red), and Centromeres (Red), and Chromatin Staining with DAPI (Blue) in a Spermatocyte at Metaphase I Two focal planes are superimposed in each picture. (A) Immunolocalization of SCP1 (red) and DAPI counterstaining (blue) of a spermatocyte in metaphase I. A small structure associated with the chromatin appears labeled (arrow). (B) Immunolocalization of SCP3 (green) and centromeres (red) on the same spermatocyte shown in (A). SCP3 appears on the three bivalents shown in these focal planes. The labeling runs on the interchromatid domain and interrupts at the chiasma sites (arrowheads). The SCP3 labeling allows the identification of the sex chromosomes (X, Y) and the DP. Sex chromosomes have already initiated their segregation and have lost contact. (C) Merge of the images (A) and (B) reveals that the structure labeled with anti-SCP1 is the DP, which in this spermatocyte has remained associated with the Y chromosome. (D) Detail of the sex chromosomes. (E) Schematic representation of sex chromosomes and the DP. Bar: 5 μm in (A–C); 1 μm in (D).

These results prompted us to analyze whether this SCP1-labeled structure may be in fact the DP. For this purpose we carried out a double localization of SCP1 and SCP3. We found that SCP1 is detectable in the DP already at diplotene (Figure 7). It first appears at discrete areas and ultimately localizes to the whole surface of the DP. During metaphase I, there is a complete correspondence between the location of the SCP3-labeled DP and the SCP1 structure associated with the sex chromosomes (Figure 8). This striking and completely unexpected result indicates that the DP undergoes a series of changes as regards its composition, and perhaps also its structure, during the end of first meiotic prophase and first meiotic division.

## Discussion

The nature of sex chromosome association in marsupial male meiosis has remained elusive for a long time. For almost forty years, from the studies of Koller [32], McIntosh and Sharman [33], and Solari and Bianchi [26], it was assumed that X and Y chromosomes in this group of mammals followed a regular pattern of recombination that ensured their proper meiotic segregation. However, when cytogenetic studies indicated the absence of synapsis between X and Y chromosomes in several marsupials [26–29], this accepted hypothesis started to tumble down. The idea that marsupial sex chromosomes do not share a region of homology has been subsequently confirmed by studies using whole X and Y chromosome paints [24,25,43]. These reports indicated that most of the marsupial Y is completely differentiated and genetically isolated from the X chromosome [25], although in some species the translocation of autosomal regions to both X and Y chromosomes has partially restored their homology [44]. In a few species these regions are specially large and they behave analogously to the eutherian pseudoautosomal regions [27,34]. Nevertheless, there are other species that even in the presence of large homologous regions, are unable to promote sex chromosome synapsis and recombination, and thus they do not perform any of the roles of the eutherian pseudoautosomal regions [27,44]. On these grounds, marsupial sex chromosomes mostly remain out of the paradigm that meiotic segregation is ensured by homologous recombination. Although some proposals have been made to explain the regular segregation of these achiasmatic chromosomes [45,46], there is a striking lack of detailed morphological studies addressing this issue.

### Sex Chromosomes Are Associated at First Meiotic Metaphase

In this paper we have carefully studied the process of meiotic sex chromosome segregation in the marsupial T. elegans by using a squash technique that maintains the native disposition of chromosomes during meiotic divisions. Our morphological approach showed that, contrary to previous reports that indicated a high frequency of sex univalents at metaphase I in the species Trichosurus vulpecula [34], X and Y chromosomes in T. elegans always appear associated during metaphase I. The separation of sex chromosomes takes place in a later stage, once X and Y are orientated to different poles, but with the autosomes still stabilized at the metaphase I plate.

This discrepancy may be due to some technical artifacts. The spreading techniques traditionally used are likely to introduce serious changes in nuclear and cell organization, disrupting the native disposition of chromosomes and their interactions with the meiotic spindle. In this sense, squashing techniques are more conservative and thus more reliable for analyzing chromosome segregation. Then, the results reported in T. vulpecula [34] could be conciliated with our findings in the sense that the univalents found in this species could be induced by the harsh treatment of spreading techniques and/or could represent the population of sex chromosomes that have already started their segregation during metaphase I. We cannot rule out the possibility that these discrepancies are due to differences between species. However, our results are consistent with those reported by Koller [32] using histological sections, who never found the occurrence of sex univalents in spermatocytes of T. vulpecula and other marsupial species.

### The DP Has a Main Role in Maintaining the Achiasmatic Association of Sex Chromosomes at Metaphase I

From our analysis on the meiosis of *T. elegans,* it seems that any attempt to explain the segregation of sex chromosomes must address two different issues: (i) how X and Y chromosomes associate in metaphase I, and (ii) why do they segregate asynchronously in relation to autosomes.

Following the currently accepted model of recombination and sister chromatid cohesion as key events to explain the association of homologous chromosomes during metaphase I, if two chromosomes in a bivalent are tied by means of one or more chiasmata, they would be able to segregate only once the cohesion between sister chromatid arms is released [12]. Several proteins have been related to the maintenance of such sister cohesion in mammalian meiosis, including the cohesin complex subunits SMC1β, SMC3, STAG3, REC8, and RAD21 [7,16–19], as well as the SCP3 protein of the SC LEs [13,15]. According to their role in maintaining the integrity of bivalents during metaphase I, these reports indicate that the onset of anaphase I is accompanied by the disappearance of these proteins along the chromosome arms.

Regarding the release pattern of some of these proteins in *T. elegans,* it is highly significant that while segregation of autosomes is associated with the loss of STAG3 and SCP3 from the interchromatid domain, sex chromosomes segregate before such release occurs. This indicates that sex chromosome association in marsupial males is not chiasmatic, thus rejecting the proposal of a recombination event between X and Y chromosomes [26,32,33].

The existence among eutherian mammals of species with achiasmatic sex chromosomes has also been reported. For these species, several mechanisms have been proposed to explain the association of X and Y chromosomes, including heterochromatin or telomeric interactions [46,47–51]. However, none of these mechanisms seems to be plausible for marsupials: first, sex chromosomes in most marsupial species do not present distal heterochromatic blocks; and second, our analysis on the distribution of telomeric sequences in T. elegans does not support the idea that telomeric DNA could be involved in the maintenance of sex chromosome association, as previously proposed [45]. Therefore, other mechanisms must be in place.

We have previously shown that the DP arises as a structure derived from the sex chromosomal AEs [30]. The development of this structure and its nature and composition seem to be common to all marsupial groups [26,27,30,31]. In this work we demonstrate that the association of the sex chromosomes by the DP is maintained in the later stages of the prophase I. Interestingly, the ends of both sex chromosomes remain in close association to the DP throughout the process of chromosome orientation on the meiotic spindle during prometaphase I and early metaphase I, and this association is lost only once the spindle forces tend to move sex chromosomes toward opposite poles. Thus, these results provide a structural support to the hypothesis that the DP has a main role in ensuring the segregation of sex chromosomes during first meiotic division in the absence of a recombination event.

### The SC-Like Organization of the DP

**Figure 9:**
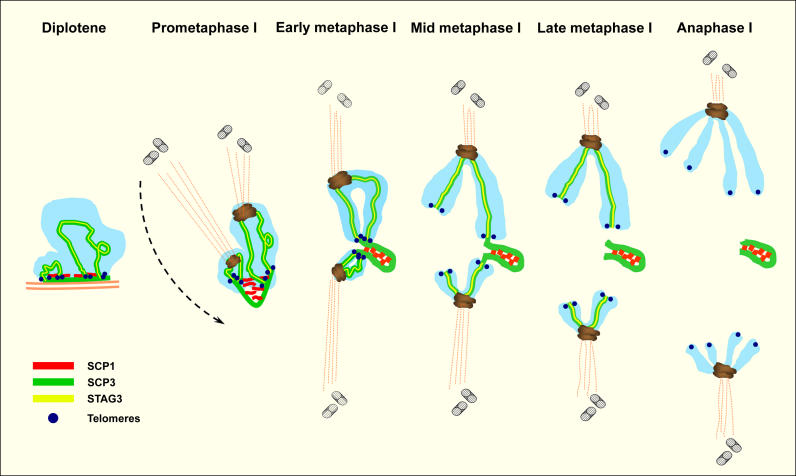
Sequence of Orientation and Segregation of the Sex Chromosomes in T. elegans Meiosis The DP is formed during pachytene as a structure derived from sex chromosomal AEs that contain SCP3. During diplotene, SCP1 is already present on the DP, where it probably associates with SCP3. During prometaphase I sex chromosomes remain associated by the DP. At this stage the DP reduces in length. We have made an interpretation of the plausible changes on the DP structure during this phase, acquiring a SC-like organization. We propose that this organization is achieved by the bending of the SCP3 outer layer, and this bending is stabilized by the formation of a SC CE-like structure composed of SCP1. The kinetochores of the sex chromosomes, as well as those of the autosomal bivalents, interact with the spindle microtubules. During this stage both centrosomes separate and by metaphase I they form the bipolar spindle. In metaphase I, sex chromosomes remain associated and are already oriented to opposite cell poles. In early metaphase I, both arms of the sex chromosomes are associated with the DP, but as soon as microtubules start to pull sex chromosomes, they begin to lose this contact. The short arms of both sex chromosomes separate from the DP during mid metaphase I. At late metaphase I the long arms of sex chromosomes definitively lose their contact with the DP and they initiate their migration to the cell poles. This segregation occurs before the disappearance of the SCP3 and STAG3 proteins from the interchromatid domain. These proteins disappear at the onset of anaphase I, allowing for the separation of sister chromatids, which remain associated only at the centromeric regions. SCP3 and SCP1 remain detectable during this stage on the DP. Therefore, the SCP3 protein present in the DP is not removed at the same time as it is removed from the interchromatid domain of all chromosomes. Perhaps SCP3 escapes from this process due to its association to SCP1. Telomeric DNA of both sex chromosomes is not associated at any stage of the first meiotic division and therefore does not play any role on their segregation.

Some of the features of the DP evolution in the late stages of prophase I are of outstanding interest. First, the DP shrinks during prometaphase I, adopting a characteristic curved shape, with the remnants of the sex chromosomal AEs immersed on it. Second, the SCP1 protein, which is the main component of the CE of the SC, incorporates to the DP from diplotene onward. This completely unexpected feature has, to our knowledge, no counterpart in any other mammal. In all species studied to date, the CE of the SC disorganizes during diplotene and then the SCP1 protein completely disappears [13,15,42]. However, in *T. elegans,* as SCP1 disappears from autosomes in diplotene, it starts to accumulate in the DP. Thus, the DP in late prophase I and metaphase I is constituted by SCP3 and SCP1, the two main components of the LEs and the CE of the SC, respectively, indicating that the DP is transformed at the end of prophase I into a SC-like structure. This hypothesis is consistent with the description made by Solari and Bianchi [26] of a “folded sheet” structure associated to the sex chromosomes in spermatocytes of the marsupial *Monodelphis dimidiata.* This folded sheet presents a tripartite structure, resembling the organization of the mature SC. The size and morphological resemblance of the folded sheet and the DP we found in metaphase I, together with the presence of SCP1 and SCP3 on it leads us to assume that they are in fact the same structure. It is difficult to ascertain at the present level of analysis how this structure is organized between the sex chromosomes. We propose that SCP1 may spontaneously assemble onto the SCP3 layer of the DP in diplotene forming a bi-layered structure. However, the DP may fold at the end of prophase I in order to form a sort of SC-like structure composed of an SCP3 outer layer and a series of SCP1 filaments in an internal position that would maintain the bending of the outer SCP3 layer (see Figure 9 for details). We think this SC-like organization does not involve the AEs of sex chromosomes, which would only make contact at the ends of the DP. We favor this model for two main reasons. First, it offers a good explanation for the structure of the “folded sheet” described in metaphase I spermatocytes of M. dimidiata [26]. Second, it explains why the DP can be released as a whole from the sex chromosomes when they initiate their migration to opposite poles, without involving DNA sequences from any of the sex chromosomes. If SCP1 were organized between sex chromosomal AEs forming a true SC, one would expect SCP1 to completely disappear upon sex chromosome separation, which is not the case. Nevertheless, other interpretations of the DP organization are also likely.

### The Many Ways to Segregate Achiasmatic Chromosomes

Although the marsupial male system may seem to be very exceptional, the presence of achiasmatic chromosomes in meiosis is a recurrent situation found in almost all groups of organisms. For any of these situations, organisms have adopted feasible strategies in order to transmit their gene content to the next generation (see [22] for review). These strategies include, among others: (i) the preservation of the SC until metaphase I, as it occurs in Lepidoptera females [20]; (ii) the development of specific structures, called segregation bodies, like those found in beetles of genus *Blaps,* which maintain the association of sex chromosomes in metaphase I [52]; (iii) the equational segregation of sex univalents in anaphase I and the association of non-sister X and Y chromatids during the second meiotic division to segregate reductionally, a process known as inverted meiosis reported in almost all hemipteran species and in some other insects [53]; and (iv) the association of homologous chromosomes through euchromatic or heterochromatic interactions, as in the case of *Drosophila* males [21,54] and the sex chromosomes in some mammalian species [46,47–51]. The case of *Drosophila* males, in which all chromosomes are both asynaptic and achiasmatic, is specially striking since recent reports have indicated that interactions between homologs are dependent on the association of specific cohesin proteins, which would be responsible for maintaining their association and thus ensuring their segregation [23].

Taken together, all these examples indicate that meiosis is a process that may offer different solutions to particular situations in order to ensure the segregation of homologous chromosomes. Interestingly, the most genuine meiotic structure, the SC, besides its role in mediating chromosome synapsis, has the ability to serve as a structure involved in chromosome segregation in organisms as different as Lepidoptera and marsupials. There are obvious differences between the solutions adopted by each of these groups. The maintenance of SC in butterfly females may just rely on a specific regulation of SC disassembly, while the development of the DP in marsupial male meiosis seems to represent a unique case of post-pachytene modification of the dynamics and organization of the SC components. However, from a functional and evolutionary point of view the possibility that the SC, or some of its components, could act as a backup system to ensure the proper outcome of meiosis when other mechanisms are switched off is really intriguing.

### Early Sex Chromosome Segregation and Implications for Cell-Cycle Progression

The precocious segregation of sex chromosomes is a feature that poses many interesting questions, not only for the processes of chromosome pairing and segregation, but also for the regulation of other cellular processes, cell-cycle progression, for instance. Since sex chromosomes in T. elegans separate before the release of sister chromatid arm cohesion, their segregation must be regulated in a different way in relation to autosomes. We suggest that a plausible explanation could be that the DP acts as a reliable mechanism to ensure the association of sex chromosomes, preventing them from separating. However, once they orientate to opposite cell poles, this association would not be strong enough to counterbalance the pulling spindle forces that tend to separate X and Y chromosomes, and they just move apart from each other (Figure 9). The fact that sex chromosome association is lost firstly at the ends of the short arms and then at the long arms indicates that the dissociation of the sex chromosomes from each other and from the DP may directly depend on the physical strength induced by the spindle forces, and not on the regulated cleavage of cohesins or other components as it occurs for autosomes. Following this model, sex chromosomes will segregate only if they are associated to opposite poles, avoiding the possibility of missegregation and ensuring that the cell transmits a complete genetic set to each of its daughters. However, the early segregation of sex chromosomes could have important consequences for the progression of the cell cycle. Since their kinetochores are precociously released from the spindle tension, they could interfere with the activation of the anaphase promoting complex and delay the onset of anaphase I, as it has been experimentally demonstrated in grasshoppers [55]. Studies in living grasshopper spermatocytes have shown that there are several possible mechanisms by which early segregating chromosomes could avoid the interference with this process [56–58]. Further studies are necessary to characterize whether some of these mechanisms are also applicable to marsupial sex chromosomes.

## Materials and Methods

Males of T. elegans Waterhouse (Didelphidae) were collected under permission in the central region of Chile. The specimens were castrated by a cut at the base of the scrotum, and the seminiferous tubules were extracted and fixed for further processing. Six specimens were analyzed. The results were completely congruent, and no individual differences were observed regarding sex chromosome behavior.

### Silver staining.

Seminiferous tubules were fixed in ethanol:acetic acid (3:1) and stored at −20 °C until further use. Then the tubules were treated as described by Rufas et al. [59]. Briefly, pieces of tubules were macerated in 50% acetic acid and squashed, the cover slip was removed after freezing the slides in liquid nitrogen and then the slides were left to dry. Afterwards, the slides were incubated in 2 × SSC (0.3 M NaCl; 30 mM sodium citrate C_6_H_5_Na_3_O_7_-2H_2_O) at 60 °C for 15 min, rinsed in distilled water, and air-dried. Finally, the slides were stained for 3 min with silver nitrate in distilled water (1/1 w/v) in a moist chamber at 60 °C, rinsed in tap water, air-dried, and mounted with Eukitt.

### FISH of telomeric sequences.

Testes were fixed in ethanol:acetic acid (3:1) and stored at −20 °C. Pieces of tubules were squashed as described above and air-dried. Afterwards, they were processed for FISH as described by Viera et al. [60]. Slides were washed twice in PBS (137 mM NaCl, 2.7 mM KCl, 10.1 mM Na_2_HPO_4_, and 1.7 mM KH_2_PO_4_, [pH 7.4]) for 15 min and then fixed for 2 min in 4% formaldehyde in PBS. Three washes in PBS for 5 min were made prior to treatment with 1 mg/ml pepsin (Sigma, St. Louis, Missouri, United States) at 37 °C for 2 min. After two washes in PBS for 5 min, slides were fixed again in 4% formaldehyde, washed in PBS, dehydrated in an ethanol series, and air-dried. Hybridization mixture containing 70% deionized formamide (Sigma), 10 μM FITC-labeled (C_3_TA_2_)_3_ peptide nucleic acid (PNA) probe (Applied Biosystems, Foster City, California, United States), 2.1 mM MgCl_2_ buffer [pH 7.0] in 8 mM Tris [pH 7.2] was added per slide, and DNA denatured by heat for 3 min at 80 °C. Hybridization was performed for 2 h at room temperature. Slides were then washed twice with 70% formamide in 10 mM Tris [pH 7.2] containing 10% BSA for 15 min, and three times with TBS (1 M Tris, 1.5 M NaCl [pH 7.5] containing 0.005% Tween-20) for 5 min. Slides were then dehydrated with ethanol, air-dried, and counterstained for 3 min with 2 μg/ml DAPI (4′,6-diamidino-2-phenylindole). After a final rinse in distilled water, the slides were mounted with Vectashield (Vector Laboratories, Burlingame, California, United States).

### Immunofluorescence.

The seminiferous tubules were squashed following the procedure described by Page et al. [61]. Seminiferous tubules were fixed for 10 min in 2% formaldehyde in PBS containing 0.05% Triton X-100. Afterwards, several pieces of the tubules were placed on a slide and squashed. After freezing in liquid nitrogen, cover slips were removed; the slides were washed in PBS for 3 × 5 min and incubated with primary antibodies.

The slides were incubated with the following primary antibodies diluted in PBS: rabbit serum A1, which recognizes SCP3 protein of the SC LEs [14] at a 1:500 dilution; rabbit serum A2, which recognizes SCP1 protein of the transverse filaments of the SC central element [42] at a 1:200 dilution; a rabbit serum that recognizes the cohesin subunit STAG3 [17] at a 1:50 dilution; and a human anti-centromere serum at a 1:100 dilution. The incubations were carried out for 1 h at room temperature in a moist chamber. Then, the slides were rinsed in PBS 3 × 5 min and incubated for 1 h with the appropriate secondary antibodies: fluorescein isothiocyanate (FITC)-conjugated goat anti-rabbit IgG (Jackson, West Grove, Pennsylvania, United States) at a 1:100 dilution, Texas Red (TR)-conjugated goat anti-rabbit IgG (Jackson) at a 1:150 dilution, and TR-conjugated goat anti-human IgG (Jackson) at a 1:150 dilution. After rinsing the slides in PBS 3 × 5 min they were stained with 2 μg/ml DAPI. After a final rinse in PBS, the slides were mounted with Vectashield.

Observations were made either on a Nikon (Tokyo, Japan) Optiphot microscope equipped with epifluorescence optics and the images were photographed on Fujichrome Provia 400*F* or Kodakchrome 100 or in an Olympus BX61 microscope (Tokyo, Japan); and the images were captured using an Olympus DP70 digital camera. All images were processed with Adobe Photoshop 7.0 software.

## Abbreviations

AE: axial element
CE: central element
DP: dense plate
FISH: fluorescent in situ hybridization
LE: lateral element
SC: synaptonemal complex
